# Functional analysis and validation of oncodrive gene AP3S1 in ovarian cancer through filtering of mutation data from whole-exome sequencing

**DOI:** 10.1186/s40001-024-01814-7

**Published:** 2024-04-12

**Authors:** Deshui Kong, Yu Wu, Qiyu Liu, Cuiyu Huang, Tongxia Wang, Zongyao Huang, Yan Gao, Yuan Li, Hongyan Guo

**Affiliations:** 1 Department of Obstetrics and Gynecology, Peking University Third Hospital, No.49 Huayuanbei Rd., Haidian District, Beijing, 100191 People’s Republic of China; 2 National Clinical Research Center for Obstetrics and Gynecology, Peking University Third Hospital), Beijing, China

**Keywords:** Ovarian cancer, Somatic mutation, AP3S1, Immune infiltration, TGF-β pathway, EMT

## Abstract

**Background:**

High-grade serous ovarian carcinoma (HGSOC) is the most aggressive and prevalent subtype of ovarian cancer and accounts for a significant portion of ovarian cancer-related deaths worldwide. Despite advancements in cancer treatment, the overall survival rate for HGSOC patients remains low, thus highlighting the urgent need for a deeper understanding of the molecular mechanisms driving tumorigenesis and for identifying potential therapeutic targets. Whole-exome sequencing (WES) has emerged as a powerful tool for identifying somatic mutations and alterations across the entire exome, thus providing valuable insights into the genetic drivers and molecular pathways underlying cancer development and progression.

**Methods:**

Via the analysis of whole-exome sequencing results of tumor samples from 90 ovarian cancer patients, we compared the mutational landscape of ovarian cancer patients with that of TCGA patients to identify similarities and differences. The sequencing data were subjected to bioinformatics analysis to explore tumor driver genes and their functional roles. Furthermore, we conducted basic medical experiments to validate the results obtained from the bioinformatics analysis.

**Results:**

Whole-exome sequencing revealed the mutational profile of HGSOC, including BRCA1, BRCA2 and TP53 mutations. AP3S1 emerged as the most weighted tumor driver gene. Further analysis of AP3S1 mutations and expression demonstrated their associations with patient survival and the tumor immune response. AP3S1 knockdown experiments in ovarian cancer cells demonstrated its regulatory role in tumor cell migration and invasion through the TGF-β/SMAD pathway.

**Conclusion:**

This comprehensive analysis of somatic mutations in HGSOC provides insight into potential therapeutic targets and molecular pathways for targeted interventions. AP3S1 was identified as being a key player in tumor immunity and prognosis, thus providing new perspectives for personalized treatment strategies. The findings of this study contribute to the understanding of HGSOC pathogenesis and provide a foundation for improved outcomes in patients with this aggressive disease.

**Supplementary Information:**

The online version contains supplementary material available at 10.1186/s40001-024-01814-7.

## Background

High-grade serous ovarian carcinoma (HGSOC) is the most aggressive and prevalent subtype of ovarian cancer, accounting for a significant portion of ovarian cancer-related deaths worldwide [1, 2]. Despite advancements in cancer treatment, the overall survival rate for HGSOC patients remains low, thus highlighting the urgent need for a deeper understanding of the molecular mechanisms driving tumorigenesis and for identifying potential therapeutic targets [3]. Recent breakthroughs in next-generation sequencing technologies have revolutionized cancer genomics research, thus offering unprecedented opportunities to comprehensively study the genomic landscape of tumors. Whole-exome sequencing (WES) has emerged as a powerful tool for identifying somatic mutations and alterations across the entire exome, providing valuable insights into the genetic drivers and molecular pathways underlying cancer development and progression [4, 5].

According to the mutational landscape of ovarian cancer and clinical trials, BRCA gene mutations exist in ovarian cancer patients with DNA homologous recombination repair deficiency [6]. Poly(ADP-ribose) polymerase inhibitors (PARPi) drugs has been developed in response to these findings and numerous clinical trials have been conducted [7]. Currently, PARPi drugs such as olaparib, niraparib and other drugs, are being clinically applied and have achieved success [8, 9]. Although the application of these newly developed drugs is becoming increasingly widespread, the 5-year survival rate for patients with epithelial ovarian cancer is less than 30% [10].

In this research article, we aimed to elucidate the genetic landscape of HGSOC via WES analysis of 90 patient samples. By analyzing the somatic mutation profile, we sought to identify key genetic alterations associated with tumor progression and patient outcomes. Moreover, we investigated the potential role of AP3S1, which is a candidate gene of interest that was identified from our analysis, in tumor immunity, as well as its prognostic significance in HGSOC. This research article provides an analysis of WES data from HGSOC patients, thus demonstrating the genetic landscape and potential clinical implications of somatic mutations. Moreover, we demonstrated the potential role of AP3S1 in tumor immunity and prognosis, which provides new perspectives on targeted therapeutic interventions for this aggressive disease. Our findings provide a foundation for personalized treatment strategies aimed at improving patient outcomes in HGSOC patients.

## Methods

### Study population

This study included 90 patients with histopathologically confirmed HGSOC (high-grade serous ovarian cancer) from Peking University Third Hospital (PUTH). We performed whole-exome sequencing on surgical ovarian cancer samples obtained from these patients. All of the patients underwent surgery and received first-line platinum-based therapy from November 2019 to December 2021. All of the selected patients underwent surgery before receiving any prior chemotherapy. The exclusion criteria for individuals included significant comorbidities, a history of tumors or tumor treatments, lack of informed consent, and metastatic ovarian cancer. Ethical approval was obtained from the Ethics Committee of Peking University Third Hospital. All of the patients provided written informed consent. Patients who were included in the study were treated in accordance with the principles of the Declaration of Helsinki and good clinical practice guidelines. We collected surgical samples from these 90 patients and conducted whole-exome sequencing following standard procedures. The TCGA data were downloaded from UCEC Xena (http://xena.ucsc.edu/). Clinical characteristic of TCGA and PUTH were performed in Table 1.

**Table 1:** Clinical characteristic of TCGA and PUTH

	TCGA	PUTH
Age		
≤ 55	161 (39.2%)	50 (55.6%)
> 55	250 (60.8%)	40 (44.4%)
Stage		
I	16 (3.9%)	3 (3.3%)
II	22 (5.4%)	4 (4.4%)
III	309 (75.7%)	52 (57.8%)
IV	61 (15.0%)	31 (34.4%)
Grade		
G1	3 (0.7%)	1 (1.1%)
G2	51 (12.5%)	1 (1.1%)
G3	344 (84.3%)	88 (97.8%)
GB	2 (0.5%)	
GX	8 (2.0%)	
Prior_treatment		
No	411 (100%)	90 (100%)
Yes	0	0
Primary_site		
Ovary	411 (100%)	90 (100%)
Other	0	0
Pathological type		
Serous cystadenocarcinoma	411 (100%)	90 (100%)
Other	0	0

*TCGA* The cancer genome atlas, *PUTH* Peking University Third Hospital

### DNA extraction

The FFPE sections of tumor tissues that were obtained from the enrolled patients were pathologically confirmed by two independent pathologists to ensure their accurate diagnosis. All of the samples were carefully selected to have a tumor cell content exceeding 20%. DNA from FFPE samples was extracted by using the DNeasy Blood & Tissue Kit (Qiagen, Inc.) according to the manufacturer's instructions. The purified DNA was quantified by using a Qubit 3.0 fluorometer (Life Technologies, Inc.) with the following qualifying criteria: extraction concentration greater than 0.3 ng/μl and a total amount greater than 10 ng. The quality of DNA was assessed by using a PCR assay on the StepOnePlus System (Life Technologies, Inc.), with the QC criterion being a Qubit/qPCR value greater than 15. All of the collected samples successfully passed the quality control (QC) step. The Accel-NGS 2S HYB DNA LIBRARY KIT (Swift Biosciences, 23,096) and HotStart ReadyMix (KAPA, KK2612) were used for library preparation and amplification, respectively. The amplified libraries were purified by using SPRI SELECT (Beckman, B23319). Somatic genomic alterations were identified from the tumor samples by excluding the germline alterations that were identified in the matched blood samples.

### Whole-exome sequencing

The amplified libraries were captured using the xGen Exome Research Panel v2 (IDT), which targets a region of 33 Mb. Finally, the samples were subjected to paired-end sequencing on the NovaSeq 6000 platform (Illumina), with a read length of 150 bp. The average sequencing depth for each sample was 500× .

### Homologous recombination deficiency analysis

The homologous recombination deficiency score (HRD score), which is also referred to as the genomic instability score or genomic scar score in some studies, was calculated by using the ‘scrapHRD’ R package. The calculation method for each HRD score has been described in previous reports and involves a combination of the number of heterozygous losses, large-scale transitions, and telomeric allelic imbalances across the entire genome [11].

### Mutation analysis

The raw sequencing data that were generated from WES were aligned to the reference human genome (UCSC hg19) by using the Burrows-Wheeler Aligner, thus resulting in binary alignment/map (BAM) files. After duplicate removal and local realignment, the Genome Analysis Toolkit (GATK) was used to identify single nucleotide variants (SNVs) and short insertions/deletions (indels). Variants were annotated by using the ANNOVAR software tool. Variants with allele frequencies exceeding 1% in each sample were selected and further annotated based on the Catalogue of Somatic Mutations in Cancer (COSMIC) database. The functional classification of each mutation followed the interpretation, reporting standards, and guidelines recommended by the Association for Molecular Pathology (AMP), the American Society of Clinical Oncology (ASCO), and the College of American Pathologists (CAP). The tumor mutation burden (TMB) for the entire exome region of each sample was calculated by using published and widely utilized methods.

### Mutation characteristics

We performed supervised analysis of mutation characteristics in the WES data using the R package YAPSA and calculated a linear combination decomposition of the mutation catalog with known and predefined features by using the non-negative least squares (NNLS) method. Mutation catalog correction was conducted by comparing the entire genome with the WES capture region to account for differences in the occurrence of trinucleotide motifs. We utilized a set of 30 publicly available mutation features, known as the AC1-AC30 (AC representing Alexandrov COSMIC), for the analysis.

### Microhomology-mediated insertions and deletions

The software SigProfilerMatrixGenerator was used to calculate MHID (microhomology insertion and deletion) in the entire exome, which explores and visualizes all types of small mutation events, including substitutions, insertions, deletions, and dinucleotide substitutions.

### Statistical analysis and bioinformatics

The R program(R-4.0.3) was used for data analysis and visualization. Statistical methods and visualization of survival analysis were performed through the Kaplan‒Meier method. Subcellular localization and immunohistochemistry data were downloaded from the HPA database (https://www.proteinatlas.org). Tumor single-cell sequencing analysis was performed via TISCH (http://tisch.comp-genomics.org/), and single-cell sequencing data were obtained from previously published literature [12]. Tumor immune analysis was performed through the TIMER database [13] and ImmuCellAI [14] based on the TCGA dataset. Functional enrichment analysis was performed with gProfiler [15]. The ‘limma’, ‘ESTIMATE’, ‘GSEABase’, ‘GSVA’, ‘survival’, ‘WGCNA’ and ‘maftools’ packages were used for data analysis and visualization. GraphPad Prism was used to analyze the data and perform the statistical analysis. All of the cellular and molecular experiments were conducted in triplicate.

### Cell culture

SKOV3 cells were obtained from the Cell Bank of Type Culture Collection of the Chinese Academy of Sciences (Shanghai, China). A2780 cells were purchased from the BNCC Corporation (Beijing, China). A2780 and SKOV3 cells were maintained in DMEM (BI, Israel). Both media were supplemented with 10% fetal bovine serum (FBS), and the cells were incubated at 37 °C in a humidified atmosphere with 5% CO2. Extensive measures were taken to ensure that all of the cell lines were free of mycoplasma contamination, and short tandem repeats (STRs) were confirmed to authenticate their identity.

### Tumor cell migration assay and wound healing assay

Transwell assays were performed by using Transwell chambers (Corning Life Sciences, Corning, NY, USA). A total of 2 × 10^5^ cells were seeded in the upper chamber of the insert. The lower chamber was filled with 700 µL of medium containing 20% FBS, which served as a chemoattractant. After incubating for 12–20 h, the cells on the lower surface were fixed with ethanol and stained with 0.2% crystal violet. The number of migrated cells in each chamber was quantified under a microscope (magnification×20), and the ImageJ software was used to determine and count the cells in the chamber.

For the scratch wound assay, a total of 2 × 10^5^ cells were plated in each well of 24-well plates and allowed to adhere overnight. Subsequently, scratch wounds were created by using 10 μL pipette tips. The cells were then incubated with serum-free medium and imaged at the same position at 0, 24, and 48 h. The extent of wound healing at each time point relative to the initial time point (0 h) was used to calculate the degree of migration. ImageJ software was used to calculate the area change of the wound.

### Western blotting

After washing with PBS three times, the cells were lysed on ice by using radioimmunoprecipitation assay buffer containing 1% phenylmethylsulfonyl fluoride (PMSF) and 1% NaF for 30 min. The cell lysates were then centrifuged at 13,362 *g* for 10 min at 4 °C. The resulting protein extracts (30–50 µg) were loaded into each lane and separated by using sodium dodecyl sulfate/polyacrylamide gel electrophoresis. Following extraction and electrophoretic separation, the proteins were transferred to polyvinylidene difluoride membranes (Millipore, Billerica, MA, USA). Subsequently, the membranes were incubated with primary and secondary antibodies. The blots were visualized by using Immobilon^®^ Western horseradish peroxidase substrate (Millipore).

In this study, the antibodies that were used for Western blotting were diluted 1:1,000. Antibodies against TGF-bβ (ab215715), p-SMAD2 (ab280888), p-SMAD3 (ab52903), VIM (ab8069), N-cadherin (ab76011), and E-cadherin (ab40772) were purchased from Abcam. Antibodies against AP3S1 (AP10499a, Abcepta) and ACTB (66,009–1-Ig, Proteintech) were obtained from Abcepta and Proteintech, respectively. AP3S1 siRNA was purchased from GenePharma. Ovarian cancer cells were subjected to protein extraction for Western blotting analysis after transfection with 80 nM AP3S1-siRNA for 48 h. An activator of the TGF-β signaling pathway known as, SRI-011381 (HY-100347, MCE), was applied at a concentration of 10 µM, and continued treatment was continued for 12 h post-transfection. The TGF-β signaling pathway inhibitor, pirfenidone (S2907, Selleck), was administered at a concentration of 5 mM, and the ovarian cancer cells were treated for 12 h. All of the cellular and molecular experiments were conducted in triplicate.

## Results

### Comparison of WES and TCGA results

**Fig. 1:**
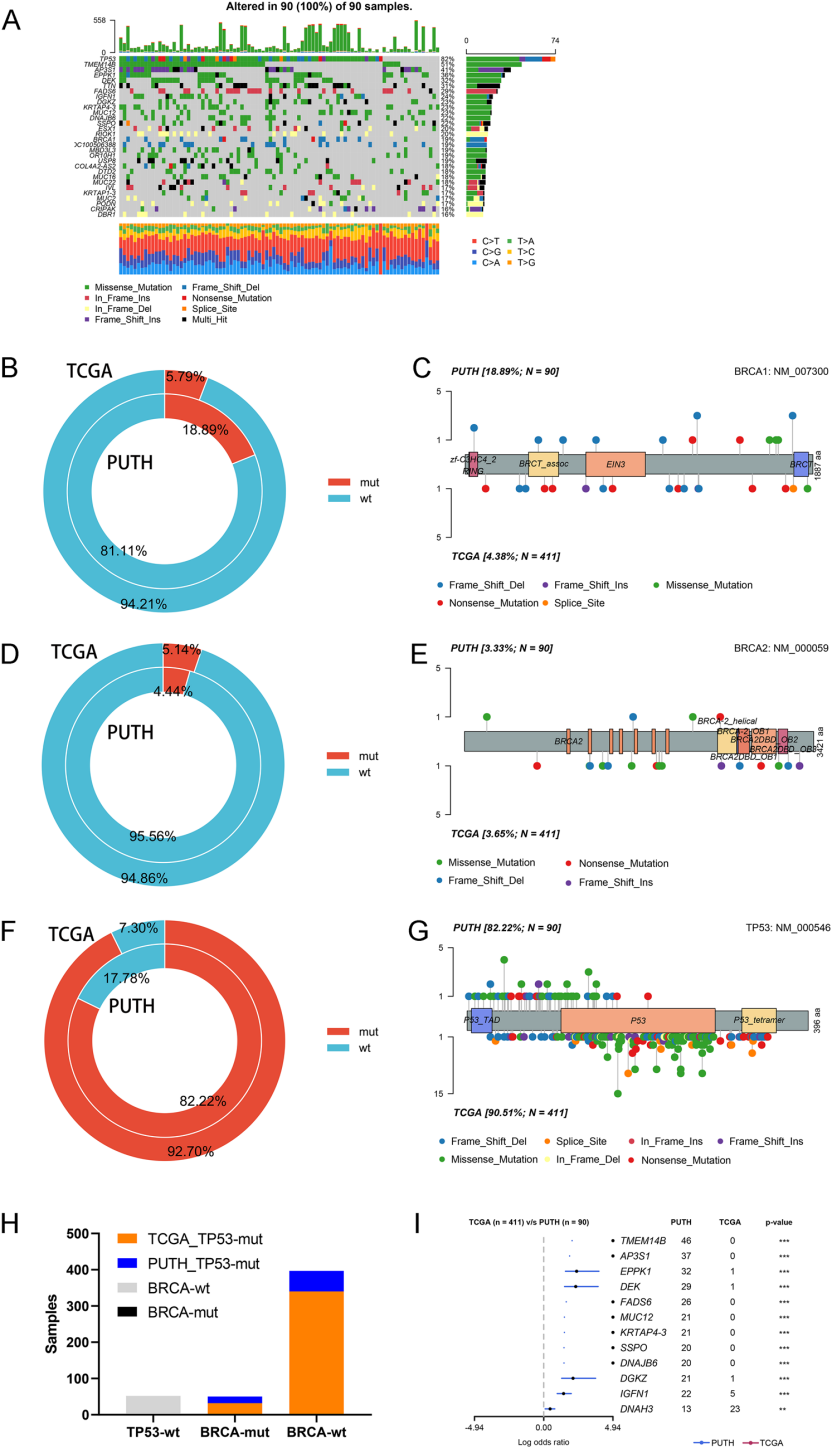
Mutations in BRCA1/2 and TP53 at PUTH. **A** Top 30 mutated genes in the PUTH data. **B** Comparison of mutation rates of BRCA1 in PUTH and TCGA. **C** Mutation sites and frequencies of BRCA1 in PUTH and TCGA. **D** Comparison of mutation rates of BRCA2 in PUTH and TCGA. **E** Mutation sites and frequencies of BRCA2 in PUTH and TCGA. **F** Comparison of mutation rates of TP53 in PUTH and TCGA. **G** Mutation sites and frequencies of TP53 in PUTH and TCGA. **H** Mutation rates of BRCA1/2 and TP53 after combining TCGA and PUTH data. **I** Forest plot comparing the differences in mutated genes between TCGA and PUTH datasets

We conducted whole-exome sequencing (WES) on 90 patients with high-grade serous ovarian cancer (HGSOC), which revealed the top 30 significantly mutated genes (Fig. 1A). In contrast to the TCGA data, wherein BRCA1 mutations were present in 5.79% of patients, our study demonstrated a higher prevalence of 18.89% in PUTH samples (Fig. 1B). The mutation sites and types that were discovered in the PUTH sequencing are shown together with the TCGA data (Fig. 1C). The mutation rates and sites of BRCA2 are also displayed (Fig. 1D–E). TP53 had a high mutation rate in both the TCGA and PUTH cohorts (Fig. 1F). The mutation status and locations are also presented (Fig. 1G). We combined the TCGA and PUTH samples and determined the overall proportions of patients with somatic mutations and wild-type TP53 and BRCA1/2 (Fig. 1H). A forest plot was generated to display the genes with the most significant differences in mutations between the TCGA and PUTH cohorts (Fig. 1I). HRD-related gene mutations in TCGA and PUTH were performed in Additional file 1: S-1A, B. A mutation word cloud based on the PUTH system mutation data is shown (Additional file 1: S-1C). The percentage of PUTH samples with BRCA1 germline mutations was 8.89% (8/90), and the percentage of PUTH samples with BRCA2 germline mutations was 12.2% (11/90) (Additional file 1: S-1D).

### Analysis of clinical characteristics and sequencing results

**Fig. 2:**
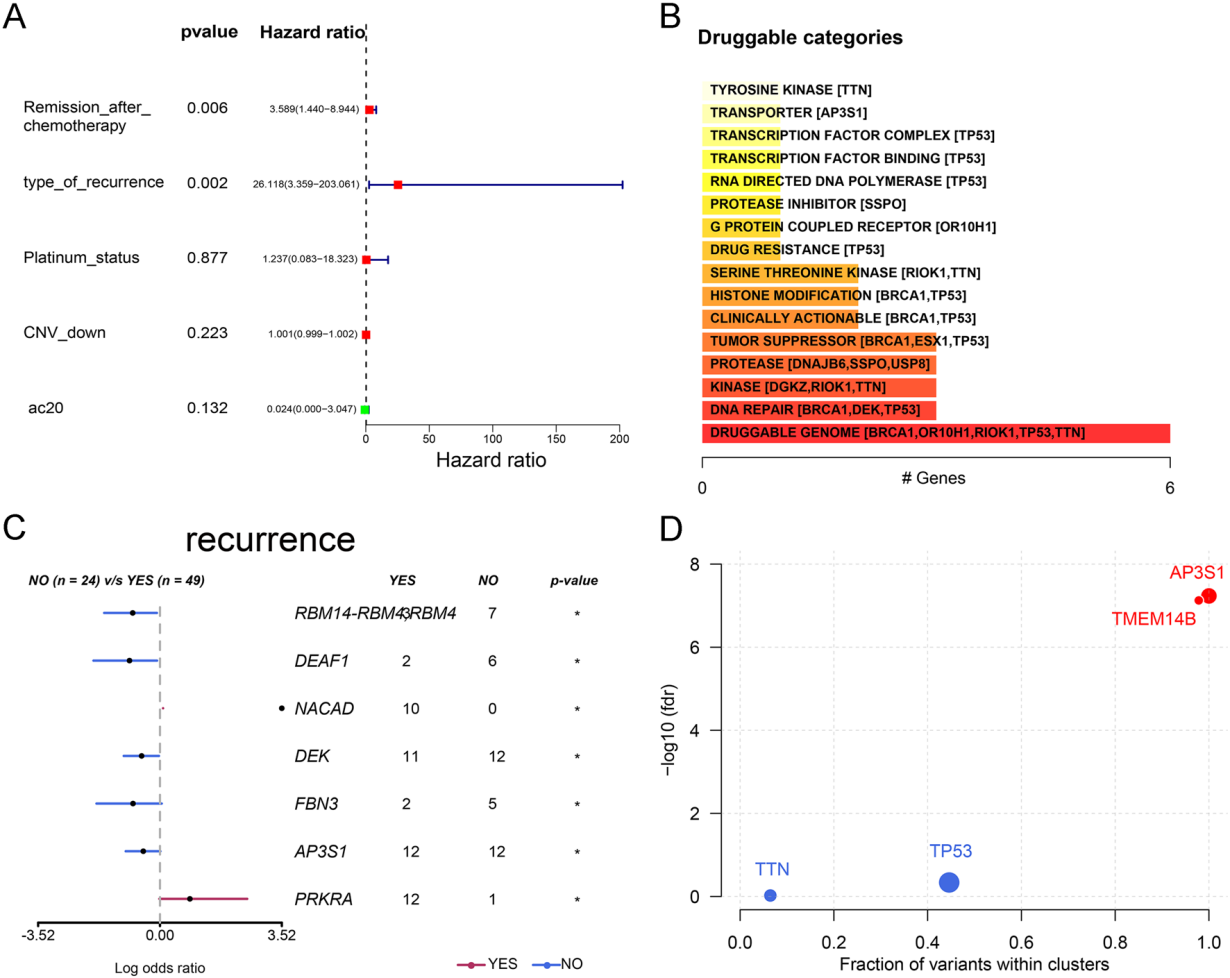
Demonstration of recurrence in patients with ovarian cancer. **A** Forest plot of clinical characteristics and recurrence outcomes in the PUTH data. **B** Drug strategies calculated based on mutation data. **C** Genes showing the most significant mutation differences between recurrence and non-recurrence samples in our follow-up patients. **D** Oncodrive genes identified from PUTH data

Cox survival analysis of progression-free survival (PFS) was performed by combining mutation data with clinical characteristics. The results showed that remission after chemotherapy and type of recurrence were closely associated with PFS (Fig. 2A). Based on the mutation data, we calculated potential druggable gene categories (Fig. 2B). A forest plot was used to compare the data from recurrent and nonrecurrent samples to identify genes with significant mutation differences (Fig. 2C). Tumor driver genes were identified based on the WES data from PUTH samples, and AP3S1 had the highest weight among these genes (Fig. 2D).

### Prognosis and expression of the oncodrive gene AP3S1 in ovarian cancer

**Fig. 3:**
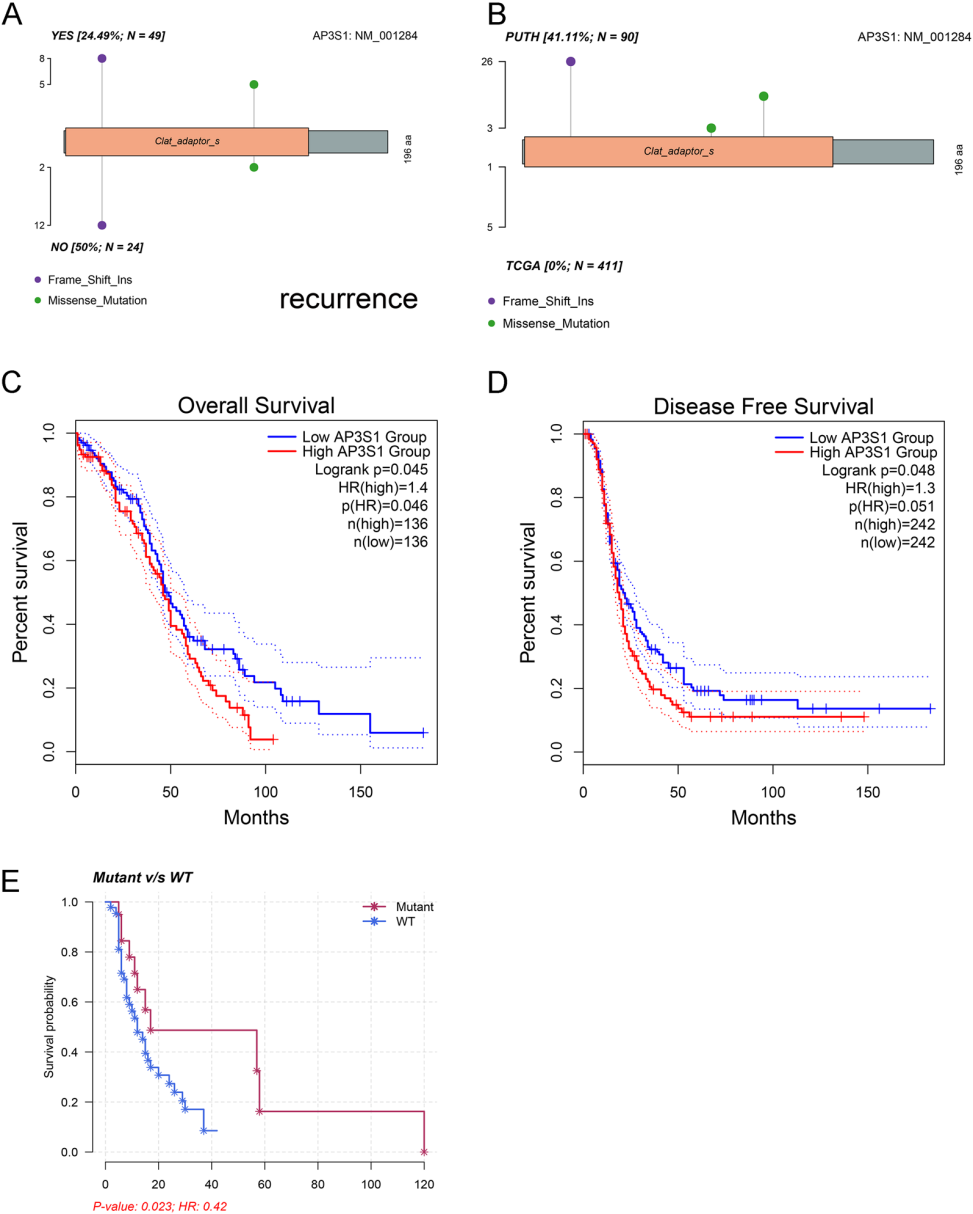
Mutational status of AP3S1 and survival analysis. **A** Lollipop plot of AP3S1 in recurrence and non-recurrence samples from the PUTH data. **B** Comparison of mutation rates and sites of AP3S1 between PUTH and TCGA data. **C** Patients with high AP3S1 expression in TCGA had worse overall survival (OS) than those with low expression. **D** Patients with high AP3S1 expression in TCGA had worse disease-free survival (DFS) than those with low expression. **E** Comparison of progression-free survival (PFS) between AP3S1-mutated and non-mutated samples from the PUTH follow-up data; patients with mutations had better PFS than wild-type patients

**Fig. 4:**
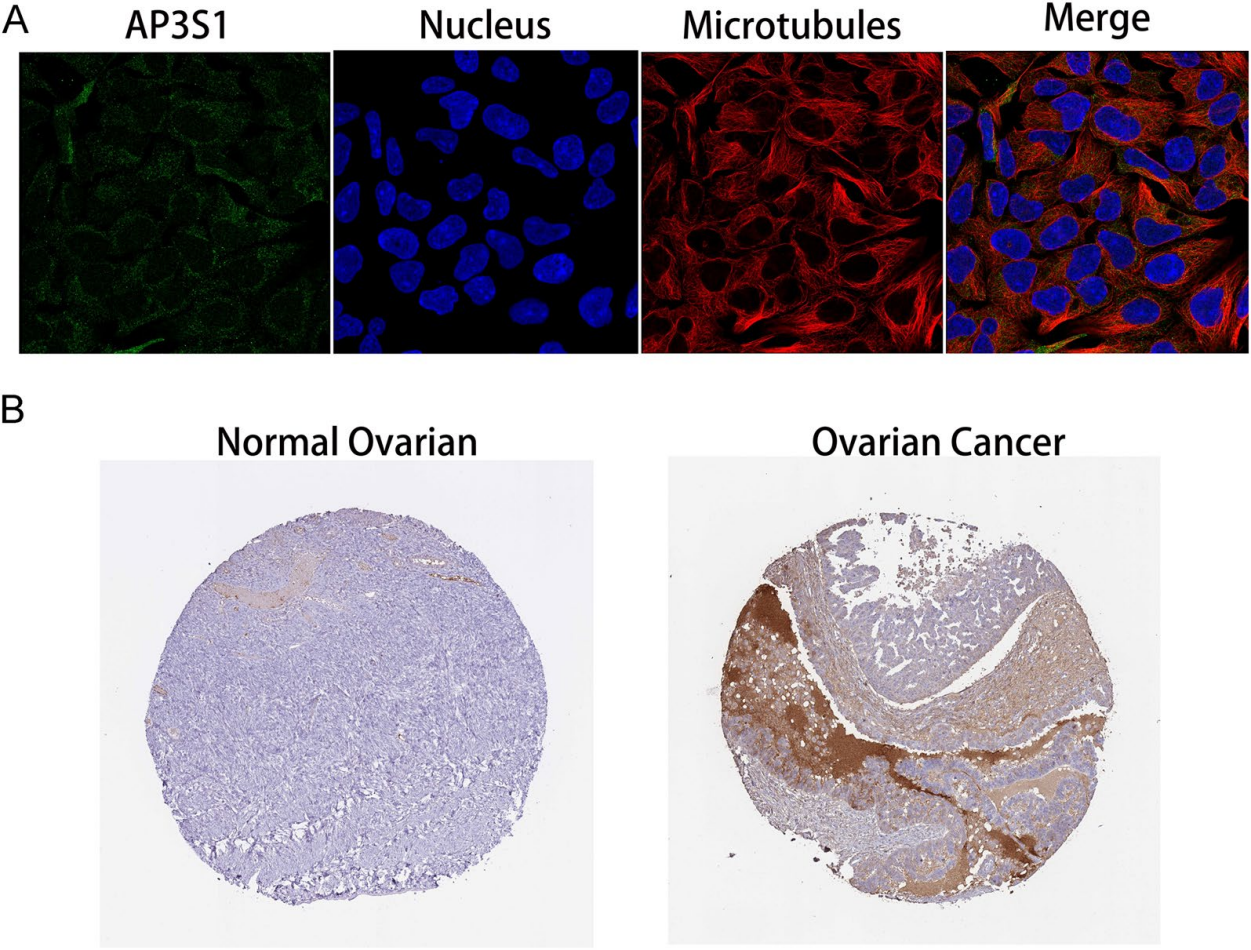
AP3S1 expression in ovarian cancer cells and tissues. **A** Subcellular localization of AP3S1 showed its predominant expression in the cytoplasm (green). **B** Immunohistochemical results indicate higher expression of AP3S1 in ovarian cancer tissues compared to normal ovarian tissues

AP3S1 had a greater mutation frequency in nonrecurrent patients according to the PUTH mutation data (Fig-3A). Interestingly, the TCGA data lacked AP3S1 mutations, which were identified in our PUTH cohort (Fig-3B). According to the TCGA data, patients with high AP3S1 expression had worse overall survival (OS) than those with low AP3S1 expression (Fig. 3C). Disease-free survival (DFS) also showed a similar trend (Fig. 3D). Our data suggested that AP3S1 mutations were associated with improved PFS in HGSOC patients (Fig. 3E). We explored the HPA database (https://www.proteinatlas.org/ENSG00000177879-AP3S1/subcellular#human, November 10, 2023) and found that the immunofluorescence results indicated that AP3S1 was mainly distributed in the cytoplasm of human cells (Fig. 4A). AP3S1 expression was possibly greater in ovarian cancer tissues than in normal ovarian tissues (Fig. 4B). This finding suggested that AP3S1 may function as an oncogene in ovarian cancer.

### Single-cell sequencing results for AP3S1

**Fig. 5:**
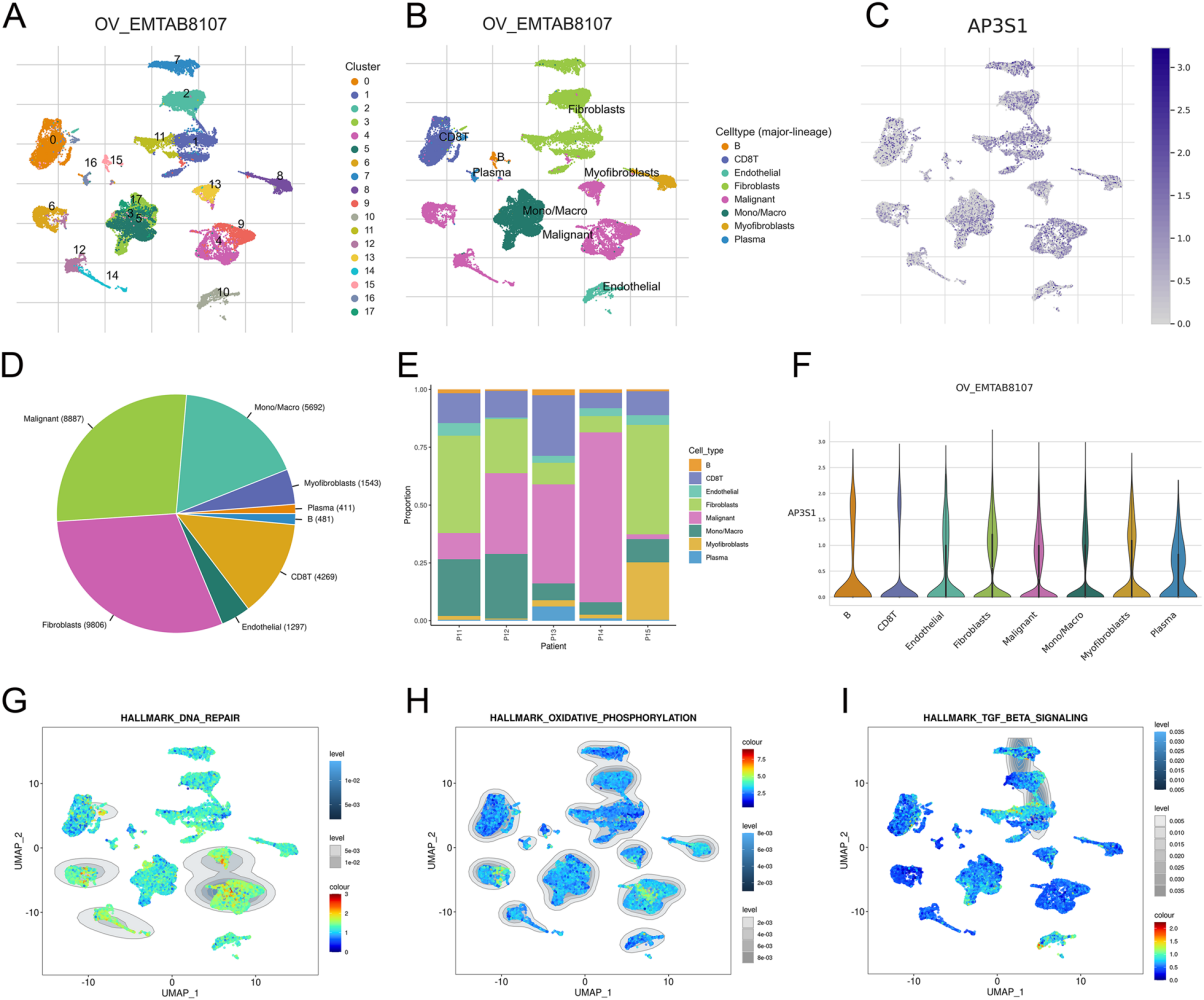
AP3S1 exhibition in ovarian cancer single-cell sequencing results. **A** Single-cell sequencing results classifying cell subgroups in ovarian cancer tissues. **B** Cell subgroups identified as immune cell clusters. **C** Expression level of AP3S1 in immune cell clusters. **D** Proportions of various immune cells. **E** Proportions of different immune cells in various ovarian cancer samples. **F** Expression of AP3S1 in different immune cells. **G** Expression of DNA repair pathways in cell subgroups. **H** Expression of oxidative phosphorylation pathways in cell subgroups. **I** Expression of TGF-β pathways in cell subgroups

We explored the single-cell sequencing results from OV_EMTAB8107 of the TISCH database (http://tisch.comp-genomics.org/) and identified the clustered cells as immune-related cells based on cell gene markers (Fig. 5A–B). We also measured the expression levels of AP3S1 in various cell types (Fig. 5C). The proportions of various immune-related cells are also displayed (Fig. 5D–E). AP3S1 expression in immune cells was relatively high in fibroblasts (Fig. 5F). The expression of genes related to DNA repair, oxidative phosphorylation, and the TGF-β pathway in this cell line is also shown (Fig. 5G–I).

### The role of AP3S1 in tumor immunity based on AP3S1 expression levels

**Fig. 6:**
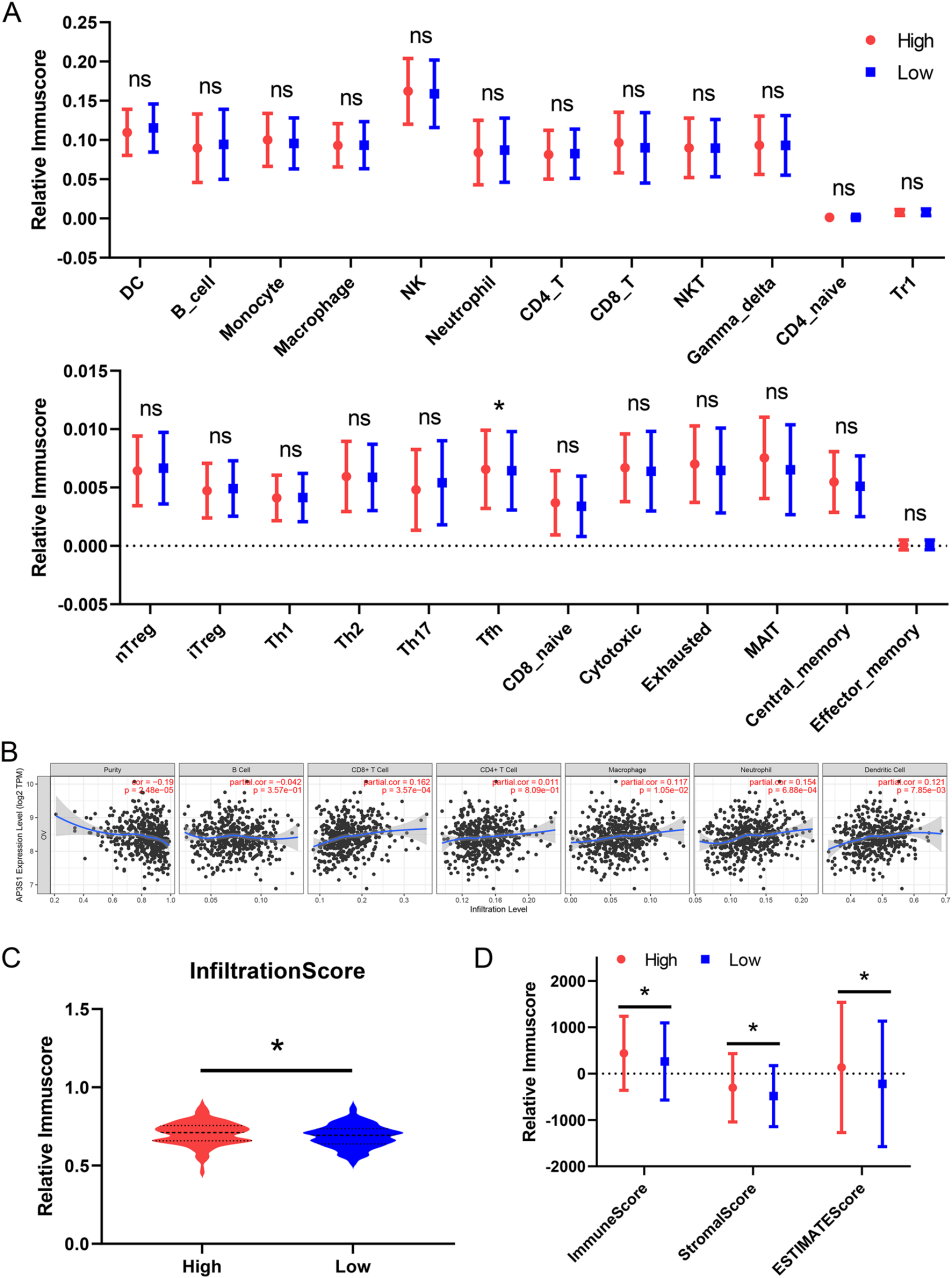
AP3S1 is involved in ovarian cancer immune infiltration. **A** TCGA samples divided into two groups based on AP3S1 expression, and the differences in infiltration of 24 immune cells between the two groups. **B** Significant correlation of AP3S1 expression in TCGA ovarian cancer samples with B cells and CD4 + T cell infiltration. **C** Significant differences in overall immune cell infiltration scores between high and low AP3S1 expression groups. **D** Significant differences in StromalScore, ImmuneScore and ESTIMATEScore between high and low AP3S1 expression groups

**Fig. 7:**
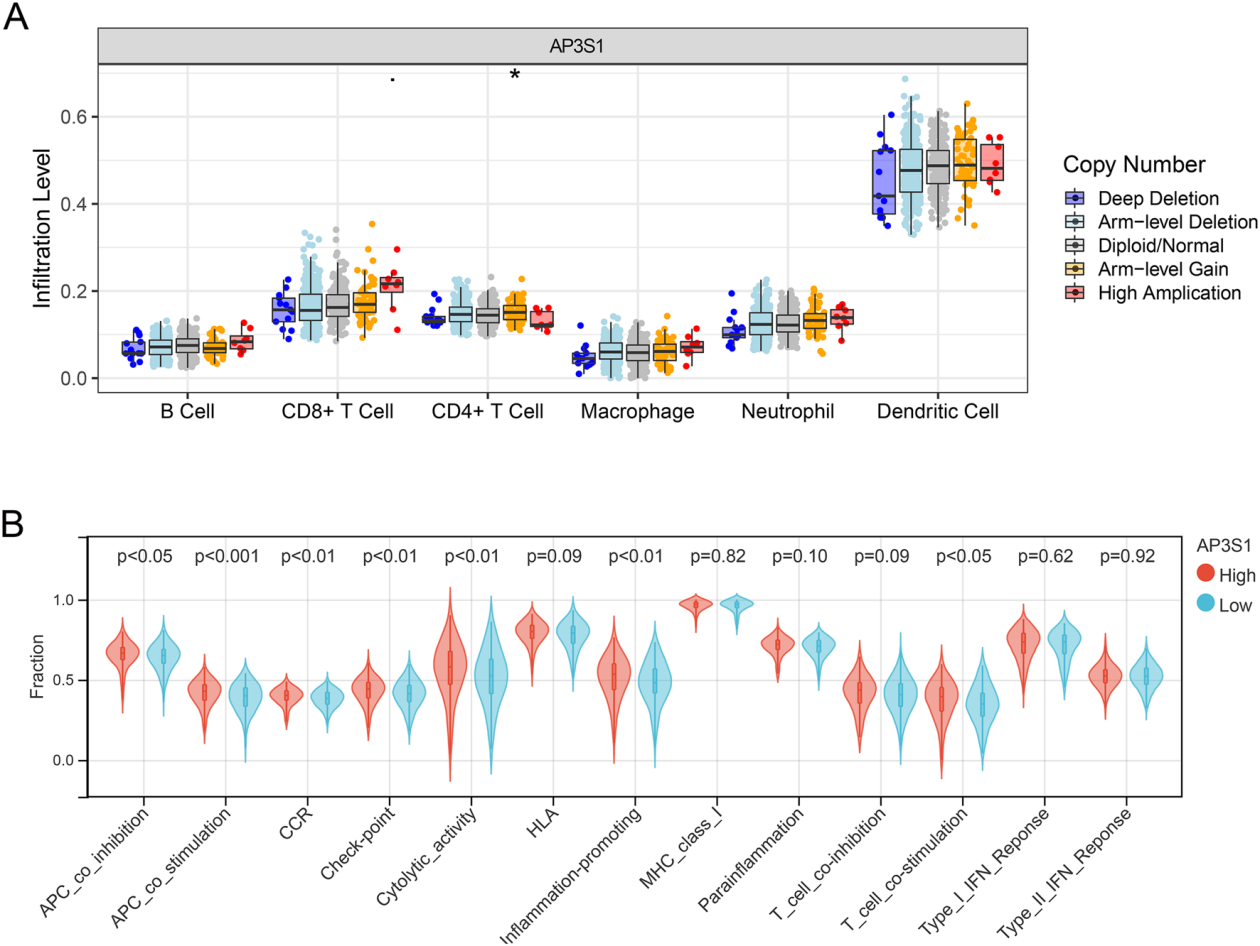
AP3S1 regulates immune cell infiltration and immune checkpoints. **A** Differential infiltration of various immune cells in different CNV types of AP3S1. **B** Comparison of AP3S1 expression groups in immune checkpoint

Based on AP3S1 expression, TCGA samples were divided into two groups, and the scores of 24 immune cells were calculated by using ImmuCellAI. The results indicated that high AP3S1 expression was associated with a high Tfh cell immune score (Fig. 6A). According to the TIMER database, the immune scores of CD8 + T cells, macrophages, neutrophils, and dendritic cells were significantly correlated with AP3S1 expression (Fig. 6B). We also used ImmuCellAI to calculate immune cell infiltration scores and found that patients with high AP3S1 expression had higher infiltration scores (Fig. 6C). Additionally, by using the ESTIMATE method, high expression of AP3S1 was associated with increased stromal score, ESTIMATE score, and immune score, whereas low expression of AP3S1 was associated with decreased immune score (Fig. 6D). We used the Timer online tool to explore the impact of different AP3S1 copy numbers on immune infiltration in different immune cell clusters. The results showed that variations in AP3S1 copy number were significantly correlated with CD4 + T cell immune infiltration (Fig. 7A). Additionally, AP3S1 was associated with differences in immune checkpoint expression, with significant differences observed in the expression of various immune checkpoint genes (Fig. 7B). These results suggest that AP3S1 may be deeply involved in the immune regulation of ovarian cancer.

### Functional enrichment analysis of AP3S1 in ovarian cancer

**Fig. 8:**
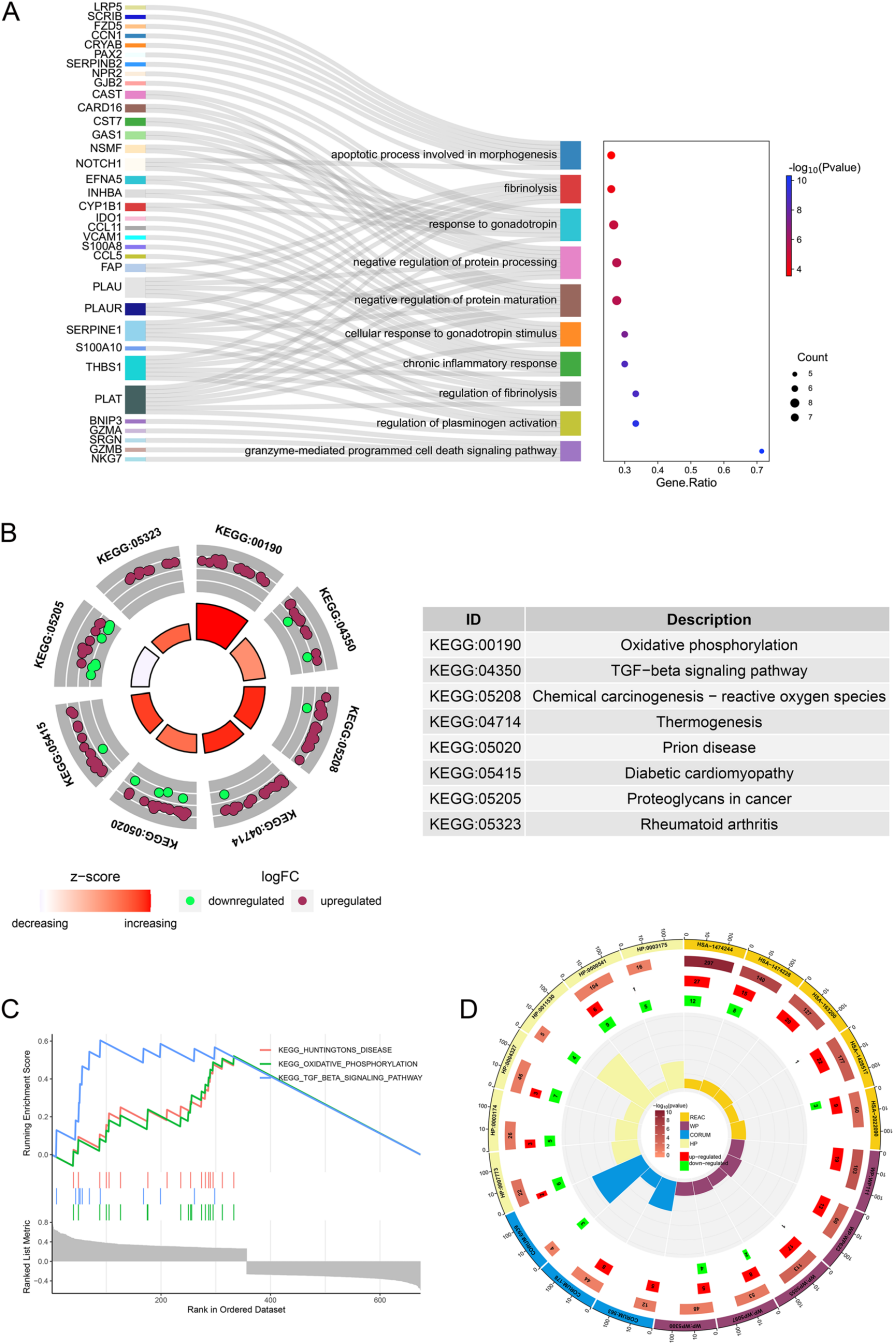
Enrichment analysis of AP3S1 in ovarian cancer. **A** Gene Ontology (GO) enrichment analysis of genes correlated with AP3S1 expression. **B** KEGG pathway enrichment analysis of genes correlated with AP3S1 expression. **C** GSEA analysis results of genes correlated with AP3S1 expression. **D** Results of Reactome, WikiPathways, CORUM, and Human Phenotype Ontology analysis of genes correlated with AP3S1 expression

We divided ovarian cancer patients into two groups based on AP3S1 expression levels and calculated the differentially expressed genes (DEGs). Functional enrichment analysis was performed on these differentially expressed genes. Gene Ontology (GO) analysis, which is a method used to categorize genes based on their function, indicated that AP3S1 was related to fibrinolysis, protein synthesis, programmed cell death, and chronic inflammation (Fig. 8A). Similarly, Kyoto Encyclopedia of Genes and Genomes (KEGG) pathway enrichment analysis, which focuses on identifying significant pathways associated with the DEGs, implicated AP3S1 in oxidative phosphorylation, the TGF-β pathway, and proteoglycans in cancer (Fig. 8B). Gene set enrichment analysis (GSEA), which is another approach for analyzing expression data, further supported the association of AP3S1 with oxidative phosphorylation and the TGF-β pathway (Fig. 8C). The results of pathway enrichment analysis for Reactome, WikiPathways, CORUM, and Human Phenotype Ontology were also displayed, which mainly suggested that oxidative phosphorylation occurs in mitochondria and retinal lesions (Fig. 8D).

### Clinical characteristics exploration and in vitro experimental validation

**Fig. 9:**
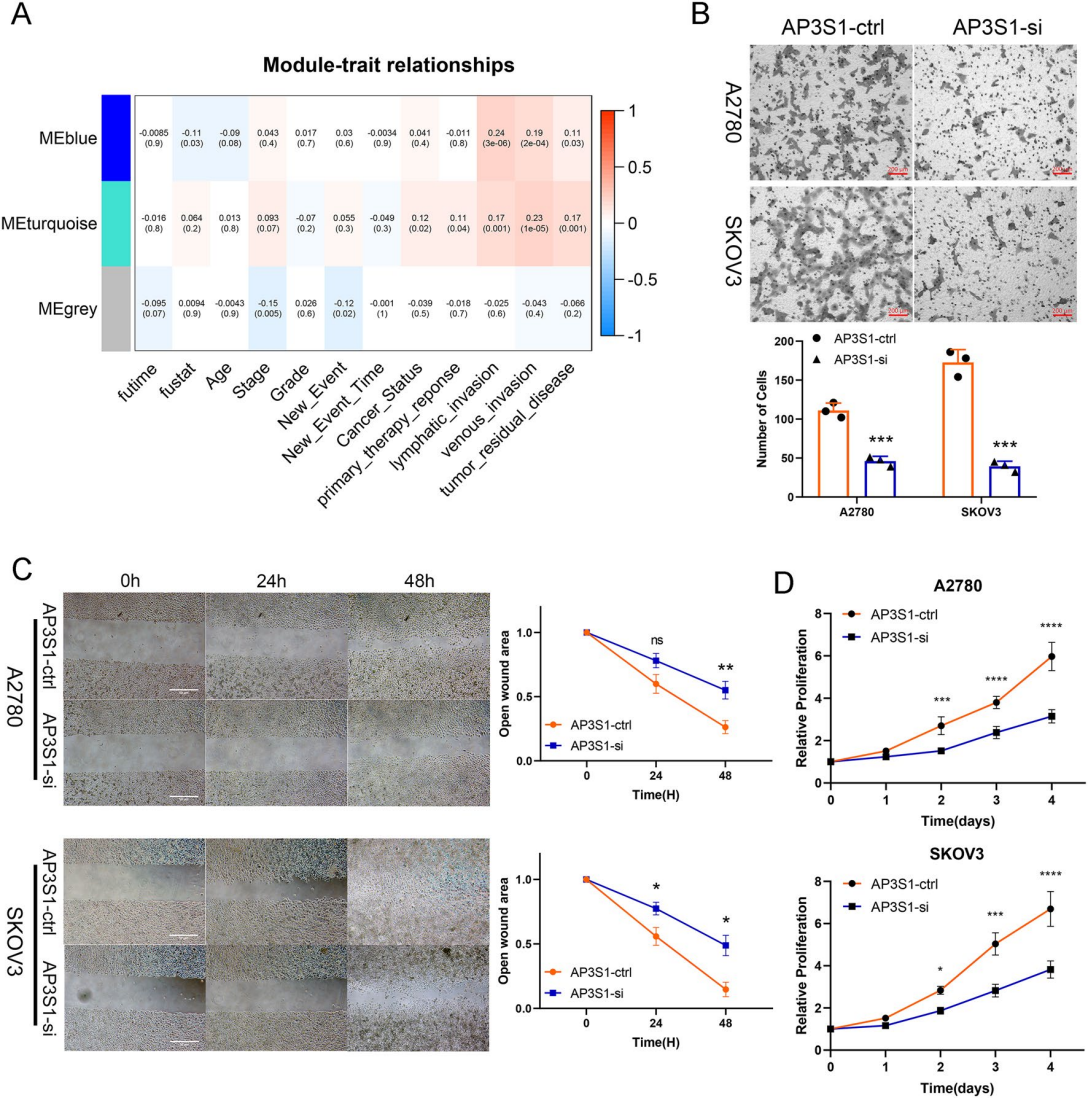
AP3S1 is involved in regulating the migratory capacity of ovarian cancer. A WGCNA analysis of genes correlated with AP3S1 expression and ovarian cancer clinical characteristic. B Significant inhibition of migration and metastasis ability in ovarian cancer cells after AP3S1 knockdown. C Scratch assay confirms that low AP3S1 expression weakens the migration ability of ovarian tumor cells. D The proliferation ability of ovarian cancer cells was inhibited after knocking down AP3S1. All data are mean ± SD. *P < 0.05, **P < 0.01, ***P < 0.001, ****P < 0.0001. Representative data are from three independent experiments

**Fig. 10:**
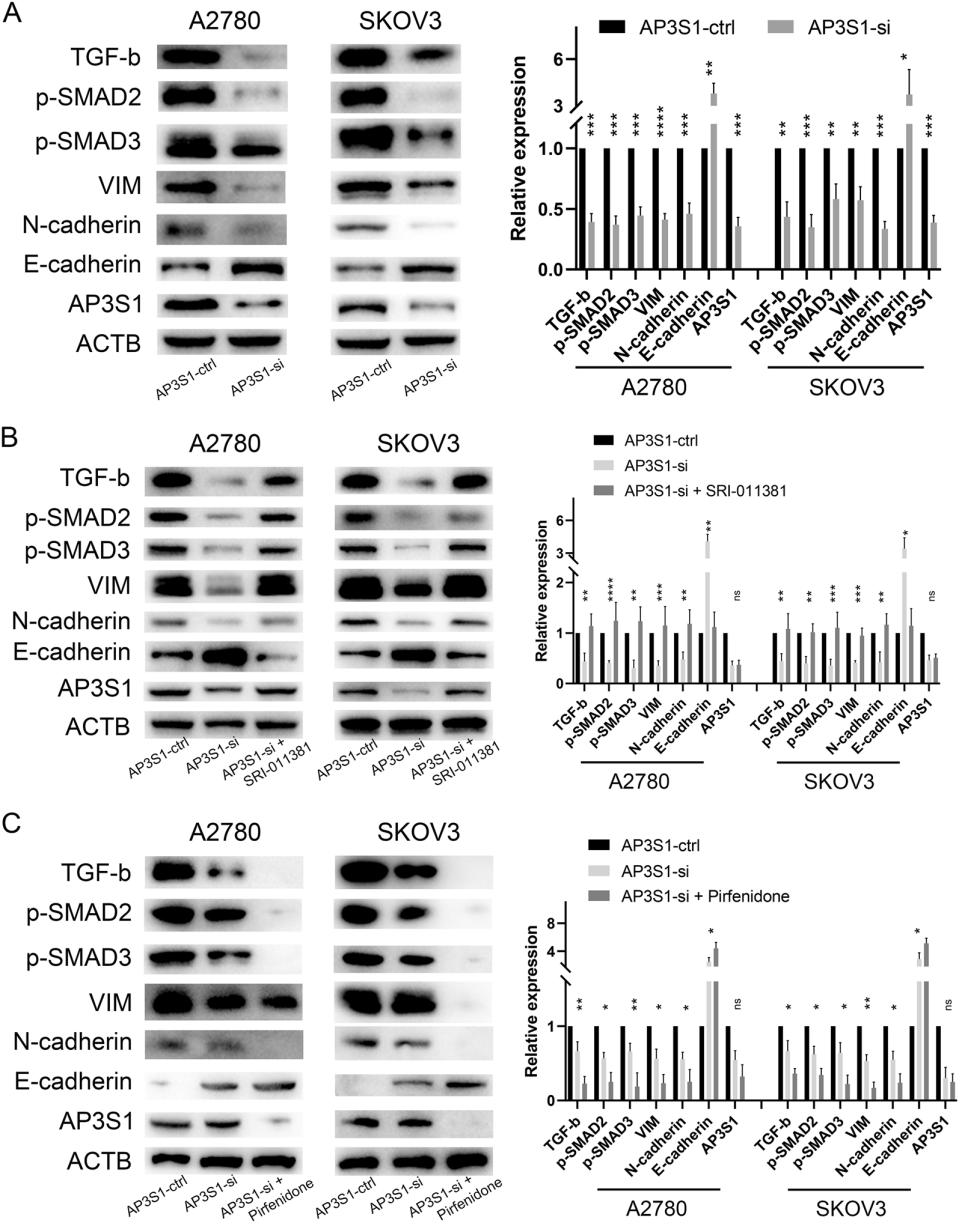
AP3S1 regulates EMT in ovarian cancer through the TGF-β pathway. A Significant inhibition of TGF-β and EMT pathways in ovarian cancer cells after AP3S1 knockdown. B The TGF-β pathway activator SRI-011381 rescues the decreased expression of TGF-β pathway and EMT-related proteins caused by AP3S1 knockdown. C Pirfenidone further exacerbates the decreased expression of TGF-β pathway and EMT-related proteins caused by AP3S1 knockdown. All data are mean ± SD. Significance calculated using the unpaired t-test. *P < 0.05, **P < 0.01, ***P < 0.001, ****P < 0.0001. Representative data are from three independent experiments

To further investigate the functional consequences of AP3S1 expression, we used weighted gene coexpression network analysis (WGCNA) to analyze the DEGs associated with AP3S1 in ovarian cancer. The results suggested that AP3S1 expression was most strongly correlated with lymphatic invasion and vascular invasion in ovarian cancer (Fig. 9A). When combining the enrichment analysis results with those of the TGF-β pathway, we hypothesized that AP3S1 may participate in the malignant phenotype of tumors by regulating the migration and invasion abilities of ovarian cancer cells. Transwell experiments were performed after AP3S1 was knocked down in A2780 and SKOV3 cells to explore the regulatory role of AP3S1 in ovarian cancer cell migration. The results showed that the migration ability of ovarian cancer cells was significantly inhibited after AP3S1 knockdown (Fig. 9B). Similar results were observed in a scratch wound healing experiment (Fig. 9C). Furthermore, knocking down AP3S1 inhibited the proliferation of ovarian cancer cells (Fig. 9D). We further investigated the underlying mechanism by which AP3S1 regulates migration. We knocked down AP3S1 in ovarian cancer cells and observed a decrease in the protein levels of TGF-β, phosphorylated SMAD2/3 (p-SMAD2/3), vimentin (VIM), and N-cadherin, whereas E-cadherin levels were increased (Fig. 10A). Interestingly, the addition of the TGF-β pathway activator SRI-011381 following AP3S1 knockdown reversed these effects, thus restoring the expression of TGF-β, p-SMAD2/3, VIM, and N-cadherin and decreasing the expression of E-cadherin (Fig. 10B). Conversely, the addition of a TGF-β pathway inhibitor after AP3S1 knockdown further decreased the expression of TGF-β, p-SMAD2/3, VIM, and N-cadherin, whereas E-cadherin expression increased even further (Fig. 10C). These findings suggest that AP3S1 promotes ovarian cancer cell migration through the TGF-β signaling pathway.

## Discussion

Although the treatment for malignant tumors has become more standardized, the mortality rate of patients with ovarian cancer remains persistently high [16]. Furthermore, the incidence of ovarian cancer is increasing, thus creating a challenging situation for gynecologists. According to the World Health Organization (WHO), it is projected that by 2025, the global incidence of new ovarian cancer cases will surpass 800,000 [17, 18]. The high incidence of ovarian cancer may be attributed to a heightened occurrence of DNA mutations in ovarian epithelial cells [19, 20]. In our study, we analyzed standardized pre-chemotherapy ovarian cancer samples through whole-exome sequencing. Our comprehensive analysis has yielded valuable insights for the treatment of ovarian cancer.

Our sequencing analysis revealed that the mutation rate of BRCA1 was greater than that reported in the TCGA cohort, whereas the mutation rate of BRCA2 closely aligned with the findings of the TCGA cohort. Furthermore, there are some differences in the mutation rate and sites of TP53 compared to those in the TCGA cohort, which was possibly due to our focus on somatic mutations rather than germline mutations. The TCGA mutation data did not distinguish between somatic and germline mutations during sequencing. To gain a holistic understanding of the mutation landscape, we amalgamated the mutation samples of BRCA1/2 and TP53, and the results were consistent with those of TCGA. According to the analysis of germline mutation data, BRCA1 and BRCA2 exhibited mutation rates of 8.89% (8/90) and 12.2% (11/90), respectively, which is similar to previously published research. By performing Cox analysis based on clinically tracked characteristics, we identified remission after chemotherapy and type of recurrence as factors correlated with PFS. The differential expression of AP3S1 in the TCGA cohort demonstrated differences in OS and PFS (Fig. 3C, D). The data from PUTH also illustrated improved PFS after AP3S1 mutation (Fig. 3E). However, the TCGA results suggested that low AP3S1 expression had a limited effect on OS and PFS in ovarian cancer patients. The PUTH data required more samples to strengthen the evidence supporting the advantageous nature of AP3S1 mutations. We will continue to follow these patients and perform additional data calculations as new follow-up information becomes available. The samples that were included in this study were surgical samples from our hospital, all of which were from Chinese ovarian cancer patients. This may exhibit limitations in a single center, and samples from multiple centers in more regions can be collected for verification in the future. The data in public databases may be biased in many aspects such as sample collection, sample processing, and release. This may affect the integrity and transparency of the data and is one of the reasons why our data differ from the data of other studies. We hope to establish more international collaborations, and researchers from multiple research centers can work together to explore and verify the mutation status of AP3S1 in ovarian cancer and its impact on clinical characteristics. Faced with the challenges of data integrity and drift, multidisciplinary collaboration is particularly important. Collaboration among experts in statistics, bioinformatics, clinical medicine, and molecular biology may be a promising direction for solving this problem.

Among the tumor driver genes derived from the mutation data, AP3S1 emerged as the most weighted oncogene. AP3S1, which is also known as CLAPS3, is a gene located on chromosome 5, that is primarily distributed in the cytoplasm, particularly in the Golgi apparatus, thus corroborating the findings shown in Fig. 4 [21]. The results from the HPA database indicate a predominance of AP3S1 in the cytoplasm rather than the nucleus. This subcellular localization might favor intracellular signal transduction. Previous studies have indicated that AP3S1 promotes tumor cell growth and invasion in lung adenocarcinoma (LUAD) [22] and serves as an oncogene in various cancers; moreover, it is potentially involved in immunosuppressive microenvironments [23]. Our findings suggest that AP3S1 may play a role in ovarian cancer recurrence. Due to the fact that no prior studies have explored the correlation of AP3S1 with ovarian cancer, our study focused primarily on investigating its role and mechanisms in ovarian cancer. Our analysis of TCGA and other collected data revealed a correlation between AP3S1 expression or mutation and the survival time of ovarian cancer patients. Immunohistochemistry results from the HPA database suggested that AP3S1 expression may be greater in ovarian cancer tissue than in normal ovarian tissue (Fig-4B). This suggested that AP3S1 could be an oncogene in ovarian cancer. Our experimental results also supported this conclusion (Fig. 10). However, more research is still needed to determine the expression and function of AP3S1 in ovarian cancer. However, more research is still needed to determine the expression and function of AP3S1 in ovarian cancer. Our data showed differences in the mutation details of AP3S1 compared to those of TCGA. We attempted to analyze the reasons underlying these discrepancies. First, this effect could be due to the smaller sample size in our dataset than in the TCGA dataset. As the sample size increases, the results of the data analysis may exhibit other variations. Second, ovarian cancer exhibits tumor heterogeneity, thus potentially leading to significant differences in sample sources between different datasets, which may result in varied data analysis outcomes. Finally, biological differences might also contribute to the effects; specifically, the patients from whom we collected samples may differ from those in the TCGA in terms of population demographics, geographical regions, environmental exposures, and genetic backgrounds, which are factors that could unpredictably affect mutation data. Through clustering analysis of single-cell sequencing data, we explored the relationships between AP3S1 expression and various immune cell clusters. AP3S1 expression varied among different immune cells. However, the small sample size and inter-individual differences might limit the stability of these findings. Based on TCGA data, we investigated the relationships between AP3S1 and various immune and stromal cells in the ovarian cancer immune microenvironment. These results suggest that AP3S1 may be associated with the expression of B cells, CD4 + T cells, and Tfh cells, and that copy number variations in AP3S1 can significantly impact the immune infiltration of CD4 + T cells. Furthermore, AP3S1 not only influences the level of immune cell infiltration, but also correlates with variations in the abundance of stromal cells. The tumor microenvironment is widely recognized as a crucial factor influencing various malignant characteristics of tumors. One of the key immunological characteristics of the tumor microenvironment is the infiltration of immune cells, which play a crucial role in tumor immune escape and the development of an inflammatory environment [24–26]. For example, in prostate cancer, thyroid hormone and androgen signaling jointly enhance inflammation and tumorigenic activation of the tumor microenvironment [27]. In addition, periprostatic adipose tissue promotes tumor drug resistance by secreting cytokines [28]. Studies in ovarian cancer have demonstrated the important role of immune cell infiltration in tumor treatment and drug resistance processes [29–31]. CD4 + T cells participate in immune surveillance by recognizing specific antigens on the surface of tumor cells [32, 33]. In the tumor microenvironment, CD4 + T cells may play a critical role in reducing immune tolerance to tumor antigens, thereby enhancing immune responses [34–36]. Our findings suggest a correlation between AP3S1 and CD4 + T cells in ovarian cancer, which provides new avenues for further investigating the role of AP3S1 in tumor immunity. The Golgi apparatus plays a vital role in various aspects of tumor immunity, including antigen processing and presentation, the synthesis of immune regulatory proteins, cellular stress responses, and tumor cell metastasis and invasion [37, 38]. The predominant expression of AP3S1 in the Golgi apparatus may be involved in these functions. Moreover, ovarian cancer tumor samples with different AP3S1 expression levels had varying immune checkpoint scores. Our research investigated the correlation of AP3S1 with the ovarian cancer tumor immune response from multiple perspectives, the corroborating prior published studies [23].

In addition to immune functions in ovarian cancer, functional enrichment analysis yielded some suggestive results, highlighting pathways such as oxidative phosphorylation and the TGF-β pathway. Existing research suggests that metabolic reprogramming is likely to occur during the development and progression of malignancies, and is one of the hallmarks of cancer cells [39]. Alterations in oxidative phosphorylation not only promote ovarian cancer cell survival and proliferation, but also enable migration, acquisition of chemotherapy resistance, maintenance of a cancer stem cell phenotype, and evasion of anti-tumor immune defenses [1, 40]. Furthermore, the TGF-β pathway has complex functions in tumor cells, whereby it acts as a tumor suppressor in the early stages of tumors and promotes tumor development in the later stages [41, 42]. The TGF-β/SMAD pathway can influence tumor cell migration and invasion by regulating EMT (epithelial–mesenchymal transition) [43, 44]. In ovarian cancer, key triggering factors for EMT include TGF-β growth factors produced by various cell types in specific tumor and metastatic environments. Although TGF-β can act as either a tumor suppressor or promoter, it exerts its pro-tumor effects, at least in part, through EMT [45]. The WGCNA results indicated that AP3S1 is associated with lymph node and vascular metastasis in ovarian cancer cells. Moreover, we validated the results of in vitro experiments and showed that AP3S1 can control the migration and invasion of ovarian cancer cells. Additionally, these experiments confirmed that AP3S1 regulates EMT in ovarian cancer cells through the TGF-β/SMAD pathway, thereby promoting tumor cell migration and invasion. However, further mechanistic studies are warranted to confirm these findings.

Our research commenced with whole-exome sequencing of ovarian cancer tissues, thus demonstrating that the mutation landscape of ovarian cancer patients in China differs from that in the TCGA database. From a gene mutation perspective, we identified AP3S1 as an ovarian cancer tumor driver gene. Through comprehensive analysis of sequencing and follow-up data, along with data from public platforms, we explored the functionality of AP3S1. In vitro experiments also confirmed that AP3S1 can regulate EMT through the TGF-β/SMAD pathway, thereby influencing the migration and invasion capabilities of ovarian cancer cells. These findings deepen our understanding of the occurrence and development of ovarian cancer, while also guiding the development of targeted drugs and clinical treatments to benefit ovarian cancer patients.

### Supplementary Information

**Additional file 1.** Mutation word cloud and information on HRD-related genes.

## Data Availability

All data generated or analyzed during this study are included in this published article [and its supplementary information files].

## Abbreviations

HGSOC: High-grade serous ovarian carcinoma
WES: Whole-exome sequencing
TCGA: The Cancer Genome Atlas
AP3S1: Adaptor related protein complex 3 subunit sigma 1
TP53: Tumor protein p53
BRCA1: Breast cancer type 1
BRCA2: Breast cancer type 2
TGF-β: Transforming growth factor beta
SMAD: Mothers against decapentaplegic homolog
PARP: Poly ADP-ribose polymerase
HRD: Homologous Recombination Deficiency
COSMIC: Catalogue of Somatic Mutations in Cancer
WGCNA: Weighted correlation network analysis
PFS: Progression-free survival
PUTH: Peking University Third Hospital
FISH: Fluorescence in situ hybridization
LUAD: Lung adenocarcinoma
