# Supplementary material for: Functional analysis and validation of oncodrive gene AP3S1 in ovarian cancer through filtering of mutation data from whole-exome sequencing
Source: Eur J Med Res. 2024 Apr 12;29:231. doi: 10.1186/s40001-024-01814-7 (PMC11015698; doi:10.1186/s40001-024-01814-7)
Supplement: Supplementary file 1 — Additional file 1. Mutation word cloud and information on HRD-related genes. [file 40001_2024_1814_MOESM1_ESM.pdf]

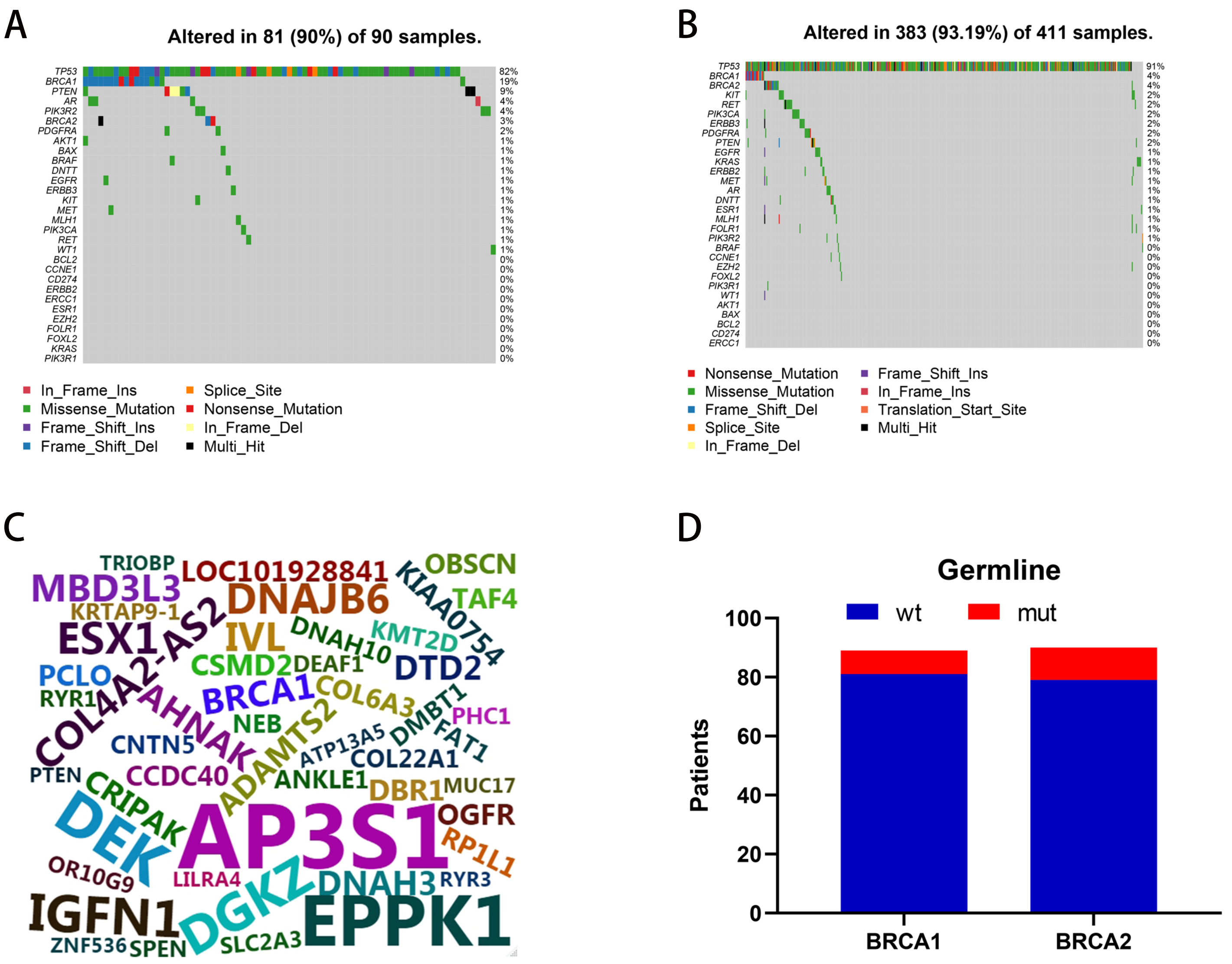

S-1 A. HRD-related gene mutations in PUTH data. B. HRD-related gene mutations in TCGA data. C. Mutation word cloud generated from PUTH data. D. Proportions of germline mutations in BRCA1 and BRCA2 in PUTH.
